# Global vaccination strategy to eradicate COVID-19: Beyond science

**DOI:** 10.7189/jogh.11.03113

**Published:** 2021-11-13

**Authors:** Mohamed Hassan Sayegh, Alaa Merhi, Hady Naal, Shadi Saleh

**Affiliations:** 1 American University of Beirut Faculty of Medicine, Beirut, Lebanon; 2 Global Health Institute at the American University of Beirut, Beirut, Lebanon; 3 Faculty of Health Sciences at the American University of Beirut, Beirut, Lebanon

Coronavirus disease-19 (COVID-19) was declared a pandemic on March 11th, 2020 by the World Health Organization (WHO). To control the pandemic, global efforts have produced promising vaccines, currently at different stages of clinical development, with 5 approved by the WHO and 14 approved by at least one country (**Table 1**). Despite continuous vaccine administration, several challenges to implementing a global vaccination strategy emerged, including limited vaccine production capacity, regulatory delays, politicization of public health interventions, resource constraints, and cultural issues. These challenges have generated increasing concerns as new strains of SARS-COV-2, the coronavirus that causes COVID-19, have emerged.

**Table 1:** Vaccines currently approved by at least one country

Vaccine	Type	Phase trials	Approval	Results published
Pfizer-BioNtech	mRNA	Completed Phase 3	Emergency use validation from WHO	Yes
			Approved in 85 countries	
Moderna	mRNA	Completed Phase 3	Emergency use validation from WHO	Yes
			Approved in 46 countries	
Oxford-AstraZeneca	Adenovirus	Phases 2 and 3 combined phases	Approved for use by WHO	Yes
			Approved in 93 countries	
Johnson & Johnson	Non-replicating viral vector	Completed Phase 3	Approved for use by WHO	Yes
			Approved in 41 countries	
Sputnik V	Recombinant adenovirus	Completed Phase 3	Approved in 65 countries	Yes
CanSino	Non-replicating viral vector	Phase 3	Approved in 5 countries	No
Novavax	Protein subunit	Phase 3	Not yet approved	No
Sinopharm	Inactivated	Phase 3	Approved in 40 countries	No

WHO – World Health Organization

Because SARS-COV-2 is a highly infectious virus with high rates of morbidity and mortality, the COVID-19 pandemic is a global problem requiring a concerted international action plan as eradication in certain areas is insufficient given the continuous appearance of multiple variants. Although the worst of the pandemic may seem to be receding in some countries, troubling signs persist in many other places, compounded with the emergence of new variants, and posing serious global risks. Few countries globally are producing COVID-19 vaccines (Australia, Belgium, Brazil, Bulgaria, Canada, China, Cuba, Denmark, France, Germany, India, Indonesia, Iran, Italy, Japan, Mexico, Netherlands, Korea, Russia, Serbia, Sweden, Switzerland, UK, USA), and only countries capable of purchasing those vaccines are doing so, while the rest have to depend on donations and support. Current national-level vaccination strategies risk marginalizing countries with limited access to vaccines, thereby exacerbating the global situation and rendering all countries vulnerable. Additionally, delays in mass vaccine administration may result in new virus mutations against which current vaccines may be ineffective, subsequently threatening present vaccination successes globally. For example, the recently identified Delta strain, which was first identified in India by early August 2021, accounted for over 83% of new COVID-19 infections in the United States (US) [1]. Conversely, faster vaccine administration and production should yield more control over variants. Therefore, it is critical that a coordinated, inclusive, and equitable global vaccination strategy is established and set into motion quickly. Contrary to the current focus on ensuring nation-level vaccine coverage, no one is protected until everyone is vaccinated, especially vulnerable and low-resource populations across the globe. In this paper, we call for the urgent formation of a high commission represented by international partners to tackle these concerns while highlighting key problematic areas on the geopolitical, economic, and cultural levels.

## GEOPOLITICS, ECONOMICS, AND CULTURAL FACTORS

Vaccination campaigns have deepened the gap across countries, now divided into vaccine-producing countries, countries capable of purchasing the vaccine but restricted by geopolitics, and developing countries unable to access vaccines. Up until August 16th, 83 percent of all vaccines distributed worldwide have been administered to individuals in higher-income countries, whereas only 0.3% of all doses have been administered to individuals in lower-income countries [2].

Vaccination discrepancies are evident across continents and across countries of the same region. Conflict-affected countries specifically are challenged by compromised infrastructures allowing black marketers to interfere for personal gains, supplying vaccines of questionable legitimacy and effectiveness. Since people demanding these vaccines may trust them, they could adopt a false sense of security and ignore preventive measures, thereby increasing the risk of spreading the virus. Developing countries may not achieve herd immunity before 2023, which may prompt people to seek above-mentioned vaccines if official channels are unable to supply legitimate vaccines [3].

**Figure:**
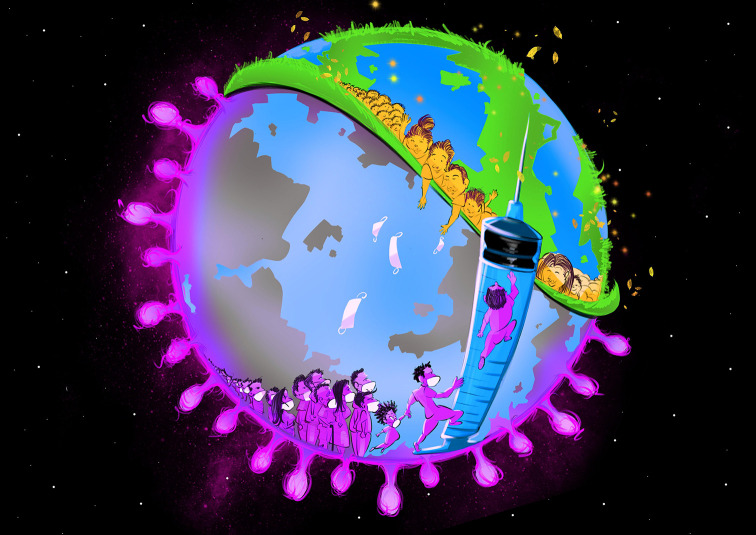
Photo: From the authors’ own collection, used with permission.

Government actions and geopolitics can also impact public perception of vaccines. For example, America’s willingness to take a vaccine declined slightly for UK-produced vaccines, while significantly dropping for Chinese ones, and almost half of Republicans refused getting vaccinated altogether due to fear and mistrust [4]. Additionally, China opened its borders for foreigners exclusively who received Chinese vaccines, which also pressured countries to offer and prioritize Chinese vaccines for frequent travelers to China [5]. Such political interferences are important predictors of national responses to public health crises.

On the level of economics, WHO should be driving a global vaccination strategy and prioritizing vaccinations in places with emerging variants and limited vaccine administration. Despite WHO creating a technology pool to encourage manufacturers to share vaccine “recipes” with Low-and Middle-Income Countries (LMICs), vaccine companies have not signed up, and governments have not supported this, their focus being on their own countries [6].

Currently, decisions about vaccine distribution globally are being made by the COVID-19 Vaccine Global Access (COVAX) facility, which is a consortium of the WHO, The Global Alliance for Vaccines and Immunization (GAVI), and the Coalition for Epidemic Preparedness Innovations (CEPI). COVAX was launched to distribute vaccines rapidly and fairly. Its initial goal is distributing 2 billion doses across at least 200 countries by end of 2021, ensuring equal access to vaccines for LMICs. However, COVAX remains underfunded with a gap of $3.1 billion after receiving US$8.6 billion in contributions [7], which is problematic since vaccine allocation through COVAX depends on their available amounts [6]. The core remaining problem is the lack of COVAX access to most effective vaccines to distribute globally and in time to effectively curtail and ultimately eradicate the pandemic.

Richer countries reportedly have oversupplies of vaccines, which undermines COVAX efforts and challenges poorer countries’ supply and distribution. For instance, Israel has been able to vaccinate 60% of its population after paying premium prices and sharing health care data with drug companies. USA ordered an oversupply of hundreds of millions of COVID-19 vaccines to be used initially within the US only until the Biden administration agreed in early August 2021 to donate and supply vaccines to lower-income countries [8]. The ONE campaign called on rich countries to offer 5% of their surplus vaccines after vaccinating 20% of their population, since many poorer countries still face uncontrollable virus spread and deaths [9]. Without modifying these strategies, LMICs are years away from vaccinating 60% of their population. Furthermore, the recent recommendations of the US administration to provide a third booster vaccine dose to prevent the recent spread of new variants may be scientifically justified but may have a negative impact on the overall vaccination strategies to eradicate the pandemic globally [10,11].

COVAX is also facing vaccine production challenges since continuous development of COVID-19 vaccines is needed, along with monitoring of new virus variants, to highlight potentially required changes to vaccines [6]. Despite the situational urgency for fair and equitable access to vaccines [7], some pharmaceutical companies used the pandemic as an opportunity to profit. Pfizer announced US$3.5 billion initial profits, and pledged to donate up to 40 million doses to COVAX, although this amount represents less than 2% of their production targets this year [12].

On the cultural level, group needs should be prioritized over individual preferences to overcome this pandemic. Religious leaders, as politicians, must proactively participate in vaccination strategies. For example, as Muslims expressed concern on whether taking the vaccine violates their fast, religious leaders refuted these claims and urged their communities to prioritize health and safety [13]. Additionally, Evangelicals were the least likely to accept taking COVID-19 vaccines given concerns about them manufactured using fresh fetal tissue, which raises vaccine hesitancy [14].

Similarly, social media contributed to vaccination roll-out strategies. It facilitated transparency and conversations despite physical distancing, such as with individuals posting about reuniting with family members, and allowed people to comfort each other by sharing their experiences of adverse events. Despite its evident success in increasing and normalizing vaccination, it also accelerated the spread of misinformation. According to a survey of 13 426 individuals globally, a strong link exists between misinformation campaigns on social media and increased negative discussions on related platforms [15]. Another survey also found that vaccines are perceived as unsafe in countries where social media was used to establish offline (on-the-ground) action [16]. Anti-vaccination efforts on social media have evidently increased vaccine hesitancy, whereby 65% of vaccine misinformation is linked to just 12 individuals [17].

## RECOMMENDATIONS

Vaccination strategies are threatened by inability to vaccinate the entire global population. Fair and equitable vaccination for all is necessary through a global vaccination strategy that needs to be established expeditiously to resolve aforementioned disparities and eradicate COVID-19. Collaborative efforts are needed by governments, health care leaders, religious figures, and social media platforms to tackle misinformation, increase accessibility, and improve vaccine uptake. Importantly, urgent action is required to reduce the proliferation of black marketers providing fake vaccination certificates to individuals who do not wish to receive the vaccine. Although some momentum has been initiated in this direction following the Biden administration offering to help in supplying two billion doses of Pfizer vaccines to LMICs, several problems persist that require further inspection [8].

First, despite these announcements, the strategy and roadmap for distribution of vaccines globally remain unclear, considering that there is no plan to prioritize who will be getting them, how will they be sent, what timeline will be followed for this initiative, which vaccines will be given, and how will administration be set. Second, a set of unanswered questions must also be tackled:

- If vaccine hesitancy remains high contrary to vaccine uptake, will vaccines be made mandatory by given nations?
- If compulsory vaccination remains ruled out by governments, should it be mandatory for certain groups and industries?
- What measures will be used to assess legitimacy of vaccines?
- Will policies be set for routine antibody testing?
- Will individuals be able to mix between vaccines?
- Will private companies soon be able to provide vaccines to the public for sale?
- How will vaccine allocation be managed in light of their expiry dates, and will policies be created to limit vaccine wastage?

With these questions in mind, and seeing that this is first and foremost a global health issue with detrimental repercussions, especially with the emergence of variants in under vaccinated populations, a clear strategy should be raised and agreed upon by international representatives. We recommend the urgent formation of a High Commission with representatives from vaccine-producing countries, rich non-vaccine-producing countries, poor countries, and the pharmaceutical industry. The primary aim of the high commission should be to transcend national boundaries and ensure equitable access to all populations to the vaccines regardless of geopolitics, socioeconomic capacity, and cultural beliefs. While there are several such high-level groups already constituted by the United Nations (UN), WHO, and a number of regional political entities such as the Group of Twenty (G20), the major problem is that they are not acting in concert, and so far, have not developed a unified global vaccination strategy with a clear goal and timetable to eradicate the pandemic. Therefore, our recommendation is the new commission should be given global authority and support to develop and execute the strategy, making sure that no country is left behind. We believe this is the only solution to eradicate this pandemic, which will also provide a working model to be replicated, adapted, and mobilized to control future pandemics.
